# Preventing sight loss from proliferative diabetic retinopathy

**Published:** 2015

The keys to preventing sight loss from proliferative DR are as follows.

1. Identify the right patients to treat with peripheral retinal photocoagulation (PRP). PRP is destruction of the peripheral retina using laser, with a minimum of 2,000 effective burns.
2. In those with established new vessels, treat them with enough PRP and, if the response to PRP is insufficient, keep going with more and more.

## When to do laser

In patients with obvious new vessels at the disc (NVD) or elsewhere in the fundus (NVE), or if there is some vitreous haemorrhage associated with new vessels, it is a straightforward decision: treat them with PRP (These are called ‘high-risk characteristics’ because there is a high risk of visual loss in the ensuing years.)

There are also benefits to treating patients with less advanced DR. The Early Treatment in Diabetic Retinopathy Study (ETDRS) showed that PRP treatment can prevent these patients from progressing to the high-risk state. In a resource-poor setting where follow-up of patients may be haphazard, or patients are unable to attend for regular appointments, treating patients with less severe disease will prevent them getting worse. The threshold which ETDRS has established for laser has become known as the 4-2-1 rule (see panel below) and equates to severe pre-proliferative DR. Patients with this level of DR and above should be treated.

**The 4-2-1 rule**

The 4-2-1 rule is:

- 4 quadrants of the fundus with dense retinal haemorrhages and microaneurysms; ***or***
- 2 quadrants with venous beading; ***or***
- 1 quadrant with intra-retinal microvascular abnormalities (IRMA).

These are all signs of retinal ischaemia, which is the stimulus for eventual new vessels – leading to tractional retinal detachment, vitreous haemorrhage and visual loss.

**When are haemorrhages and micro-aneurysms dense?**

**Figure 1.**
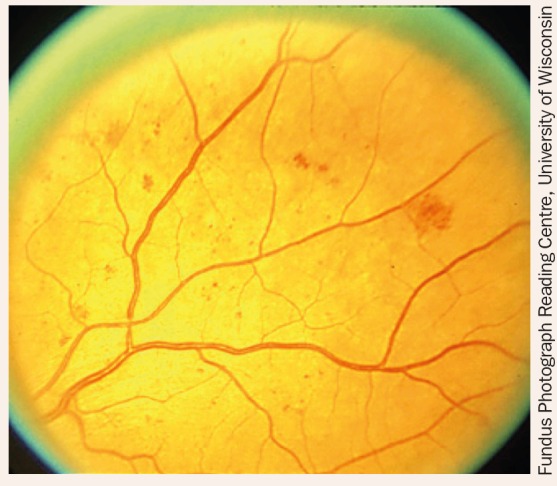
ETDRS Standard 2A. Retinal haemorrhages and microaneurysms at this density in all four quadrants indicate severe preproliferative retinopathy, an indication for peripheral retinal laser

The ETDRS study referred to standard photographs (Figure 1), if these are not available a rule of thumb is 5 or more haemorrhages and micro-aneurysms in a 1 mm wide slit, wherever in the quadrant you place the slit with a 90D lens.

**Venous beading** is clear dilation of retinal venules with accompanying constriction so it looks like a string of sausages.

**IRMAs** are abnormal branching, or network of vessels within the retina. ETDRS used standard photographs to define the size of IRMA that was significant. In a low- or middle-income country setting, with unreliable follow up, any definite IRMA warrants laser treatment.

**Cotton wool spots** are also a sign of retinal ischaemia and tend to occur in the border between well-perfused and poorly perfused retina. Although not part of the threshold, cotton wool spots are important in gaining an assessment of retinal ischaemia particularly in the absence of fluorescein angiography. They can push the clinician towards laser treatment.

Remember, these signs tend to occur together, so where there are cotton wool spots or dense haemorrhages or micro-aneurysms, look closely for IRMA or venous beading.

## Tips for successful laser

It is important that, before you start, the patient knows what to expect and what the aims of treatment and potential side effects are. In particular, you should stress that the laser treatment is to prevent visual loss in the future and is not intended to improve vision. At the start of the treatment, titrate the strength of the laser burn and adjust the laser power to achieve a visible burn which is not too harsh or bright white.

**Remember:** doubling the duration or power doubles the fluence (laser energy delivered per square millimetre), whereas halving the diameter increases the fluence by a factor of 4.

**Figure 2.**
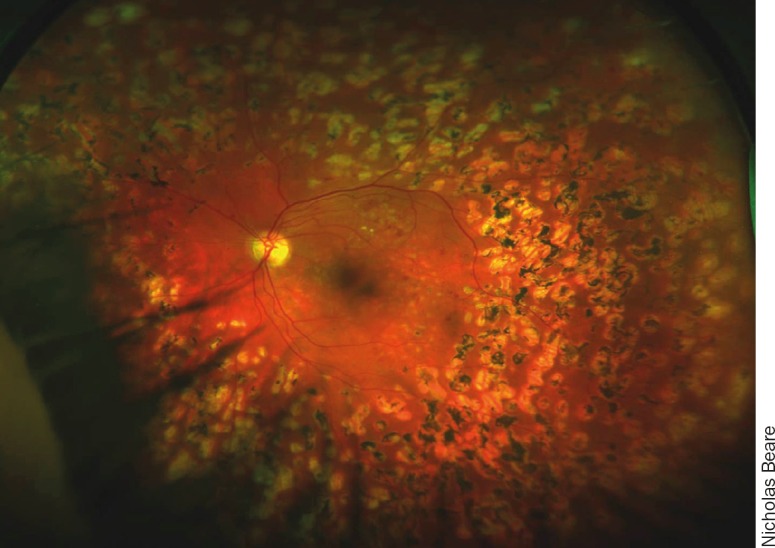
Wide field fundus photograph showing peripheral retinal laser (PRP) and macular laser (most evident on the temporal macula). There is potential to do more PRP just nasal to the disc

Modern spot sizes are smaller than in the ETDRS era, 200 microns being the standard. The duration of each laser burn is also shorter, and I recommend 0.02 s (20 ms). This reduces the laser injury and you do not have to worry about lasering over retinal vessels with this short duration. I start with 200 mW laser power if there is a clear lens. In patients with lens opacity, more power is required. Increase the power in 50 mW steps until a burn is visible. If it is too harsh (it will appear white, with a sharply defined edge), turn the power down in 25 mW steps (Figure 2). The central retina is thicker than the peripheral retina, so if you start centrally, you will have to reduce the power as you treat more peripheral retina.

The temporal quadrant is often under-treated and a zone of significant ischaemia, as it is a watershed between the vascular arcades. The laser should be brought up to the temporal edge of the macula, approximately 2 disc diameters from the foveal centre. It helps to define this border temporally with laser burns and work progressively peripherally away from it to avoid inadvertent macular coverage, or worse, a foveal burn. The clinician should know where the macula is at all times.

**NOTE:** If during the treatment you have lost visible burns with the fluence unchanged, it is usually either a focusing issue or loss of coupling gel in the contact lens. Pause, detach the lens, refill with gel and continue.

To treat pre-proliferative disease, 2,000 to 3,000 effective burns are usually sufficient, particularly if you are relatively certain that the patient will return for an examination and further treatment if needed. With proliferative DR, more burns may be required.

Call patients back for a follow-up visit to see if the new vessels regress over the following 3–6 months. If they are not regressing, more treatment is needed. In this scenario, you should treat between the original burns, up to 500 microns from the nasal disc edge and within the arcades, with two or three burn rows and as far into the peripheral retina as you can reach with your lens. Around 5,000 burns maybe required.

White fibroglial tissue will not disappear, but you are aiming to get the vascular component to regress. However, the longer the new vessels have been present (often associated with glial tissue), the harder it will be to get them to regress completely. It is okay to accept incomplete regression if the situation is stable, and you have done as many burns as you think is reasonable.

If there is vitreous haemorrhage it is very important to apply as much laser treatment as possible, as quickly as possible, whilst there is a view. There may be a small vitreous haemorrhage with scope for laser, before a larger one obscuring your view prevents any treatment. Where there is a small vitreous haemorrhage, laser is therefore urgent-because any subsequent and more severe haemorrhage is likely to have a much better outcome if the DR had been treated before the haemorrhage occurs.

## Complications and side effects of peripheral retinal laser (PRP)

PRP inevitably sacrifices some peripheral retina, butin most cases this does not have any effect on vision. In about 10% of cases, patients notice a reduction in visual field or night vision.

The effect on night vision may be more noticeable in low-income countries, where night vision is essential. The more PRP is required, the more likely this is to bean issue and it may affect the person's ability to drive. This is a trade-off with preserving any vision at all.

A foveal burn, affecting central vision, is possible but should not happen if the operator makes sure where the macula is at all times, and only switches the equipment from ‘standby’ to ‘treat’ when she or he is ready to start lasering.

Macular oedema can be induced by an aggressive and extensive PRP session. This can damage the patient's confidence by making the vision worse afterwards. If possible PRP should be offered in two treatment sessions of about 1,500 burns each to avoid this. If necessary, macular laser should be applied before PRR, or at least at the same time as the first session if the PRP is urgent.

## Role of antiVEGF in PDR

Intra-vitreal anti-VEGF injections such as bevacizumab (Avastin) only buy time until more definitive treatment. One special situation in which they might be of benefit is where severe ischaemia has led to rubeotic glaucoma. An injection of bevacizumab can induce regression of new vessels, reduce IOP, improve pupil dilation and allow laser application. Bevacizumab can be used just prior to vitrectomy to reduce bleeding during surgery and make it technically easier. However, new vessels may recur aggressively if definitive laser treatment is not commenced within a month.

## Surgery for proliferative DR

Vitrectomy surgery has limited availability, particularly in sub-Saharan Africa. Early treatment with laser should reduce the need for vitrectomy, however patients will inevitably present late particularly with vitreous haemorrhage resulting in sudden visual loss. This is the commonest indication for vitrectomy in DR. Where there is any view of the fundus, PRP is indicated. When there is not there are two scenarios: the patient who has had previous PRP and the patient who has not. If a patient has previously had a complete PRP then you can afford to wait and the haemorrhage will usually clear. There may be traction on a non-regressed new vessel but active neovascularisation should not be progressing. Where a patient has had no previous PRP the disease is actively progressing behind the haemorrhage. Here an early vitrectomy is indicated if available, usually with pre-treatment by an injection of anti-VEGF. If vitrectomy is impossible, then you can monitor and apply laser to visible zones as soon as there is any clearing.

The other indication for vitrectomy in PDR is progressive tractional retinal detachment (TRD) affecting the macula. TRD elsewhere does not require surgery. Unfortunately the eventual outcome is often poor because of the accumulated damage to retinal function, including ischaemic maculopathy.

